# Hypothesizing Balancing Endorphinergic and Glutaminergic Systems to Treat and Prevent Relapse to Reward Deficiency Behaviors: Coupling D-Phenylalanine and N-Acetyl-L-Cysteine (NAC) as a Novel Therapeutic Modality

**DOI:** 10.23937/2378-3656/1410076

**Published:** 2015-12-17

**Authors:** Kenneth Blum, Marcelo Febo, Claudia Fahlke, Trevor Archer, U Berggren, Zsolt Demetrovics, Kristina Dushaj, Rajendra D. Badgaiyan

**Affiliations:** 1 Department of Psychiatry, University of Florida College of Medicine, Gainesville, FL, USA; 2 Division of Nutrigenomics, LaVita RDS, LLC, Draper, UT, USA; 3 Division of Applied Clinical Research & Education, Dominion Diagnostics, LLC, North Kingstown, RI, USA; 4 Division of Neuroscience-Based Therapy, Summit Estate Recovery Center, Los Gatos, CA, USA; 5 Department of Psychology, University of Gothenburg, Gothenburg, Sweden; 6 Department of Clinical Psychology and Addiction, Eotvos Lorand University, Budapest, Hungary; 7 Division of Clinical Neurology, PATH Foundation NY, New York, NY, USA; 8 Departments of Psychiatry & Behavioral Sciences, Keck School of Medicine of USC, Los Angeles, CA, USA; 9 Department of Psychiatry, University at Minnesota, Minneapolis, MN, USA

## Introduction

**Figure 1:**
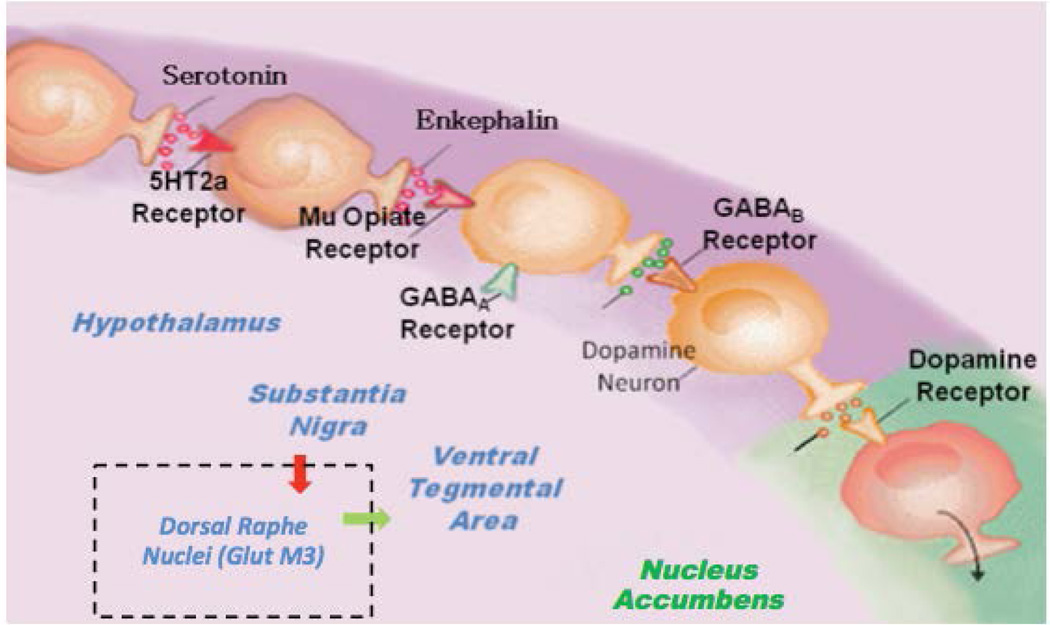
Brain Reward Cascade (BRC)

In this hypothesis, we are proposing that the combination of D-Phenylalanine and N-acetyl-L-cysteine (NAC) - two substances that have never been utilized together - is an important advancement to treat Reward Deficiency Syndrome (RDS) [1]. The key motivation for this notion is that the combination will have synergistic attributes to induce dopamine release as well as dopamine stabilization at the brain reward circuitry via different mechanisms. We hypothesize that preferential release of Dopamine, for example, at the Nucleus Accumbens (NAc) along with glutaminergic homeostasis induces not only the release, but also the appropriate regulation of dopamine function that could lead to required “Dopamine Homeostasis.” One way to explain this premise is to review the Brain Reward Cascade (BRC) developed by the one of us (KB) along with Gerald Kozlowski (Blum & Kozlowski 1989) [Figure 1] [1]. The BRC highlights the mechanism in which the proposed D-Phenylalanine and NAC combination works.

Through this particular cascade, the hypothalamic serotonergic system is stimulated, which causes stimulation of delta/mu receptors by serotonin and further enkephalin release. Initiation of the enkephalinergic system prompts a block on GABA transmission at the substantia nigra via enkephalin stimulation of GABA neuron mu receptors. GABERGIC activity is impacted by endocannabinoid and glutamate receptors. This inhibition of GABA transmission permits any slight changes in GABA activity. These changes allow for dopamine release at the anticipated region of the NAc (with permission [1]).

Understanding the BRC provides the rationale for the hypothesis that the combination of D-Phenylalanine and NAC may be an effective RDS treatment. Since GABA is an inhibitory transmitter that fine tunes dopamine release at the VTA-NAc, it is a key target to control dopamine regulation. For example, if there is high GABA activity, the result will be a lower dopamine release at known reward sites (NAc) leading to a lack of well being, which is then linked to drug-seeking behavior. On the other hand, if GABA activity is low, then possibly too much dopamine is released at the NAc, leading to psychosis. This mechanism is essential in terms of treating all RDS behaviors by regulating GABA activity.

For over 40 years, it has been recognized that the Dorsal Raphe Nucleus (DRN, classified as a serotonergic structure) and the Ventral Tegmental Area (VTA, classified as a dopaminergic structure) are two of the more relevant brain reward areas, where electrical stimulation produces response at the highest rates and lowest thresholds (meaning, very sensitive). Although multiple studies have examined both the DRN and VTA and its contribution to reward, these studies have been focused on only serotonergic effects on reward. As a result, these investigations have produced conflicting results, and the true role of DRN to VTA circuitry in regulating motivated behaviors is still unknown. Contrary to the widespread idea that the major input from DRN to VTA is serotonergic, Qi et al. [2] found that DRN neurons expressing the vesicular glutamate transporter-3 (GluT3) are the major input from DRN to VTA. Within the VTA, these DR-GlutT3 neurons mostly develop synapses on dopamine neurons; some of these dopamine neurons, as Morales [3] found, specifically innervate the NAc. By genetic approaches to specifically express rhodopsin in channel DR-GlutT3 neurons, it was also found that intra-VTA light stimulation of the VGLUT3-fibers elicits AMPA-mediated excitatory currents on dopamine neurons that innervate the NAc. Such stimulation causes dopamine release in the NAc, reinforces instrumental behaviors, and establishes conditioned place preference. Morales et al.’s [3] discovery of a rewarding excitatory synaptic input to the meso-accumbens dopamine neurons by a glutamatergic projection arising selectively from neurons of the DRN that contain VGLUT3 suggest that new targets may be important to boost motivation in the RDS patient. Moreover, unpublished work from NIDA (the Morales group) also found that GABA from the substantia nigra induces regulation of the VGLUT3 neurons and as such, fine-tunes the release of dopamine from the VTA to NAC.

## D-Phenylalanine (DPA)

Accordingly, we know that D-Phenylalanine (DPA) is an inhibitor of the enzyme (enkephalinase-a carboxypeptidase) known to breakdown (catabolize) endorphins, especially enkephalins. Thus, if we increase brain enkephalins by administering DPA, the amount of enkephalins will increase in the brain as previously reported [4].

Specifically, as observed in one study [4], when D-Phenylalanine is administered for 18 days in alcoholic C57/blk mice, endorphin levels increased in the pituitary and striatum and altered the genetically disposed alcohol-seeking mice to significantly lower their alcohol consumption to those levels seen in mice who dislike (or avoid) alcohol, much like the DBA mice. This finding published in *Alcohol* [4] specified the foundation for the function of enkephalinase inhibition as a therapeutic aim in alcoholism treatment as well as the first United States patent for an amino-acid therapy following several court appeals.

While this effect translates to a reduction in alcohol-craving behavior, it has never been shown to actually increase dopamine release at the NAc by itself. However, the mechanism is clear, and in combination with other precursor amino-acids and other substances, it has been shown via neuroimaging studies to enhance not only resting state functional connectivity, but also connectivity volume (recruitment of neuronal firing in the brain reward pleasure site), especially in abstinent heroin addicts [5].

## Development of KB220Z

The development of KB220Z occurred soon after the first report on the enkephalinase inhibitor D-phenylalanine in 1987 [4]. KB220Z is a neuroadaptagen technology developed to decrease or eliminate increased propensities for addictive behaviors such as psychostimulant and alcohol abuse and food cravings, amongst others. KB220Z promotes healthy dopamine function as well as regulation of pleasurable desires. The design of the KB220Z complex follows the brain reward cascade with the final intent of facilitating DA release throughout the reward circuitry. In a study on abstinent heroin addicts, KB220Z causes increased neuronal firing between the NAc and brain structures responsible for cognition, such as the dorsal hippocampus and anterior thalamus [5].

While we have not yet determined the actual release of DA, we are planning to use a single scan dynamic molecular imaging technique to understand the nature of DA release in the human and animal brains following administration of KB220Z. This technique allows detection, mapping, and measurement of DA released endogenously following a pharmacological or behavioral challenge [6]. Using this technique, we have previously observed dopamine release in some of the same areas where enhanced connectivity was observed in the current experiment.

We believe that these robust and selective results are due to inhibition of γ-aminobutyric acid (GABA) transmission in the substantia nigra through serotonergic-opioidergic-glutaminergic interactions reducing inhibitory control over DA release throughout the reward network [7]. In support of these findings, we found similar results in humans showing enhanced regulation of dysregulation (widespread theta) in the cingulate gyrus one hour following administration to abstinent psychostimulant addicts by increasing alpha and low beta (β) waves [8]. Preliminary research from this laboratory using fMRI in abstinent heroin addicts compared to placebo one hour after administration of KB220Z also showed activation in the NAc and reduced hyperactivity in the putamen [5]. Importantly, this finding of increased areas of activation could have therapeutic significance, especially in light of the reduced brain grey matter volume during cocaine administration to humans as reported by Bell et al. [9] It is plausible that KB220Z, due to inhibition of COMT (via rhodiola *rosea*), resulted in higher DA in the synapse and as such, enhanced DA activity [10].

## Characteristics and Neuropharmacology of KB220 Variants

KB220Z is composed of several ingredients in confirmed, evidence-based consumption levels as follows: Thiamine, 15 mg (1033% of Daily Value); Vitamin B6, 10 mg (500%); Chromium polynicotinate (ChromeMate^®^) 200 mcg (166%); dosage of amino acids and herb combination named Synaptose™, comprised of DL-Phenylalanine, L-Tyrosine, and Passion Flower Extract; Metalloglycoside™ Complex including Arabinogalactans, N-Acetylglucosamine, Astragalus, Aloe Vera, Frankincense Resin, White Pine Bark Extract, and Spirulina; and Rhodiola (RhodiGen™; L-Glutamine; 5-Hydroxytryptophan (5-HTP); Thiamine Hydrochloride; Pyroxidal-5-phosphate; and Pyridoxine HCl. Kb220Z is manufactured by Cephram, Inc. (New Jersey) [11].

In unpublished rodent studies from our laboratory, it was observed also in images of individual rats and was unrelated to the presence of gross motor artifacts that would add an artificial correlative structure to the results. In fact, maps correspond to resting-state connectivity for the left NAc. Above placebo, KB220Z increased connectivity, especially between left-right accumbens, dorsal striatum, and limbic cortical areas, such as the anterior cingulate, prelimbic and infralimbic regions. Correlation maps for representative subjects presented at a threshold between 0.35 ≤ z ≥ 1.2 [12].

Moreover, there was a significant increase in functional connectivity of NAc with medial and lateral anterior thalamic nucleus and surrounding somatosensory cortex. Thus, it appears that treating rats with KB220Z increased connectivity within the reward system. The treatment also appears to have resulted in the recruitment of additional brain regions outside the reward system, potentially including these within an integrated network. The reason for this recruitment is unclear, but likely leads to the emergence of behavioral features connected to the corresponding functions of individual structures. In this rodent study, connectivity volume was assessed by applying a correlation threshold value of z = 0.3 to all subjects and quantifying the volume above this threshold. Voxels were then converted to mm3 based on 3D voxel resolution. Increased connectivity volume was shown in select brain regions including the following: nucleus accumbens, mediodorsal thalamus, infralimbic cortex, dorsal hippocampus (D), anterior cingulate cortex, and somatosensory cortex. Significantly different from the placebo, t ≥ 3.4; p ≤ 0.05 with multiple comparison correction was assessed by using the Holm-Sidak method. Consequently, KB220z containing D-PA increases both resting state functional connectivity as well as connectivity volume in a default network involved in drug seeking behaviors [12].

These data illustrate the modulatory actions of a putative dopamine agonist (KB220Z) upon resting state functional connectivity (rsFC) in association to a key region of the reward system: the NAc. We find that KB220Z increased connectivity between this central striatal reward structure and areas of the brain that are critical for cognitive processing: the dorsal hippocampus and anterior thalamus. These brain regions were also consistent with changes observed in metabolic activity following optogenetic stimulation of the rat NAc using small animal positron emission tomography (PET) and [18F] deoxyglucose (FDG) [13]. These results are the first to show such an effect. However, there are recent human studies that have examined the role of DA in rsFC, which when taken together with the present novel finding, may provide additional insight [5].

One such study examined rsFC following challenges with levodopa (L-DOPA) or haloperidol [14]. The L-DOPA treatment resulted in increased functional connectivity in many regions, including the NAc and temporal-parietal areas as we observed in rats treated with KB220Z. Interestingly, administering methylphenidate (which elevates extracellular DA levels) to non-abstinent cocaine abusers lead to region-specific decreases and increases in the strength of connectivity (neuronal firing), where striatal regions were observed in becoming less connected or having less neuronal firing, while cortico-cortical and cortico-limbic regions showed greater connectivity (increased neuronal firing). The findings may very well improve the use of KB200Z in conjunction with other DA treatments.

## Understanding balancing the endorphinergic and glutaminergic systems to treat RDS

The importance of these previous studies (above) is to provide a mechanism whereby DPA acts through stimulating both the delta and mu opiate receptors by enhanced brain enkephalins leading to a gentle blockade of GABA transmission, which causes a physiological release of dopamine at the brain pleasure site. Acceptance of this fact, coupled with a secondary, but equally important mechanism involving glutaminergic stimulation via NMDA receptors and subsequent action of GABA transmission at the substantia nigra, should represent a synergistic mechanism to the provider BRC regulation and Dopamine homeostasis. It is interesting that many believe that the glutaminergic system when dysregulated results in an inhibition of GABA neurotransmission and increases Dopamine release [15]. It is our premise that while some have suggested that increased Dopamine release leads to drug reinstatement, we are proposing otherwise in terms of long-term treatment of RDS and promotion of regulated dopamine function, not inhibition.

However, we also believe that since individuals with risk for all RDS behaviors may have genetic polymorphisms in the glutaminergic system leading to reduced activity and less GABA inhibition, it is noted that by using DPA to increase brain enkephalins, it seems useful to provide a mechanism for balancing the glutaminergic system to prevent an overload of extracellular dopamine, instead of the appropriate amount that could lead to dopamine homeostasis. On this basis, and never combined prior to this current thinking, the addition of N-Acetyl cysteine (NAC) should induce glutaminergic homeostasis and overcome any abnormal amount of dopamine being released at the NAc allowing for enhanced ability to achieve “dopamine homeostasis”. NAC treatment should enhance overall RDS treatment combinations.

## N-Acetyl Cysteine (NAC) and its Treatment Uses

The effects of a cysteine-glutamate transporter enhancer on the neurochemical and behavioral effects of cocaine and amphetamine in nonhuman primates have been investigated by Bauzo et al.[16] It was hypothesized that augmenting extrasynaptic glutamate release with NAC, a cysteine prodrug, would diminish cocaine- or amphetamine-prompted surges in extracellular dopamine and their related behavioral-stimulant and strengthening results [16]. However, in non-human primates, unlike studies in rodents and clinical trials in humans, NAC did not alter the behavioral stimulant effects of cocaine or amphetamine or self-administration of these addicting compounds [17].

Although all the clinical studies are preliminary and utilized relatively small sample sizes, the fairly stable anti-addictive properties of NAC postulate convincing proof that this medication, along with other composites that reestablish glutamate homeostasis, could possibly demonstrate to be successful pharmacotherapeutic support in the management of RDS when combined with DPA [18]. In fact, NAC used alone has failed [19].

The NAc is a part of the brain found in the vertebrate basal forebrain and it has been implicated in drug addiction. Several neurotransmitters involved in NAc circuitry have been associated with different issues related with drug addiction, such as compulsive consumption and relapse. In particular, the glutamate system has been associated primarily to relapse after elimination of drug-seeking. The dopamine system has been associated largely to compulsive drug consumption [20]. The glutamate homeostasis hypothesis focuses on the relations of synaptic and extra synaptic levels of glutamate, and their influence from the prefrontal cortex (PFC) to the NAc circuitry [3].

Following repeated drug consumption, there is a surge in the deregulation of homeostasis, which causes glutamate release from the Pre-Frontal Cortex (PFC) to the NAc throughout relapse. Glial cells also play a central part in this hypothesis, where glial cells form the connections between the PFC and the NAc via changing glutamate levels in both synaptic and extra synaptic areas. On the other hand, cocaine self-administration and withdrawal causes growth of the surface expression of subunit glutamate receptor 1 (GluA1) of alpha-amino-3-hydroxy-5-methyl-4-isoxazolepropionic acid (AMPA) receptors at the NAc level. Cocaine self-administration and withdrawal also stimulate the development of subunit glutamate receptor 2 (GluA2), deficient of Ca2+permeable AMPA receptors (CP-AMPARs) at the NAc level. Antagonism of the CP-AMPARs decreases desires [17].

It is accepted that cocaine and morphine stimulate alterations in dendritic spine morphology by adjusting actin cycling [21]. These alterations are comprised of a primary surge in spine head diameter and growth in AMPA receptor expression, trailed by a second phase of spine head diameter retraction and decrease of the AMPA receptors’ expression in spines. Aside from glutamate and dopamine, other elements such as brain-derived neurotrophic factor (BDNF), can affect NAc activity and produce changes in dendritic spine density [21]. BDNF also stimulates drug behaviors like self-administration and relapse. Neither apoptosis nor neurogenesis is responsible for the neurobiological activities subjacent to adult cocaine addiction (rodent or human) [21].

## Other Therapeutic Drugs

Various therapeutic drugs such as N-acetyl cysteine (NAC), modafinil, acamprosate, and topiramate have been confirmed in preclinical and/or clinical models for easing drug relapse [22]. Furthermore, these therapeutic drugs aim at the glutamatergic circuitry between the PFC and the NAc. NAC and acamprosate have exhibited unpredictable results in clinical trials. Modafinil and topiramate have shown little success, but much more clinical trials are needed. Based upon the present findings, it is suggested that further exploration of therapeutic methods be done, including focus on synergism between various drugs and neurotransmitters The inconsistency in the results of some therapeutic drugs (preclinical versus clinical trials) could be associated with the limited examination of preclinical models that imitate polydrug abuse patterns (e.g., cocaine with alcohol). The arrangement of polydrug consumption is a marvel of substantial incidence at the clinical level.

Extra synaptic glutamate exhibits dopamine function regulation in the mesocorticolimbic pathway, which plays a significant part in psycho stimulant behavioral pharmacology [23]. Glutamate basal levels are mostly regulated by the cystine-glutamate transporter and deliver glutamatergic tone on extra synaptic glutamate receptors. It is essential to understand that too much glutaminergic activity will stimulate an undesirable hypodopaminergic result [24]. The balance between endorphinergic and glutaminergic systems will stimulate “dopamine homeostasis” in the long run.

## Failed Uses of N-Acetyl-L-cysteine (NAC)

The outcomes of a cystine-glutamate transporter enhancer on the neurochemical and behavioral results of cocaine and amphetamine use in nonhuman primates have been studied. It was theorized that enhancing extra synaptic glutamate release with N-acetyl-L-cysteine (NAC), a cystine prodrug, would offset cocaine- or amphetamine-stimulated surges in extracellular dopamine and their analogous behavioral-stimulant and reinforcing effects. In vivo, microdialysis was used to assess cocaine-induced alterations in extracellular dopamine (DA) in the caudate nucleus (n = 3) [16]. NAC considerably reduced cocaine-induced surges in dopamine, but had variable results on amphetamine-induced surges in dopamine (n = 4). Individual subject groups were also trained on a fixed-interval plan of stimulus expiry (n = 6) or on a second-order plan of self-administration (n = 5) to differentiate the behavioral-stimulant and reinforcing results of psycho stimulants, respectively. Systemic administration of NAC did not modify the behavioral-stimulant results of either cocaine or amphetamine. Additionally, cocaine self-administration and reinstatement of formerly terminated cocaine self-administration were not changed by pretreatment with NAC [16]. Thus, drug interactions on caudate neurochemistry in vivo were not revealed in behavioral measures in squirrel monkeys. The current outcomes in nonhuman primates do not help the use of NAC as a pharmacotherapy for cocaine abuse, though rodent and clinical studies indicate otherwise.

Other compounds that may be added to enkephalinase inhibition for dopamine release include, but are not limited to sodium nitroprusside (SNP) [25], which accounted for the late SNP-induced dopamine (DA) increase in dialysates from the striatum of freely moving rats. Nonetheless, this compound by itself has not been studied as an anti-craving substance. It is our suspicion, however, that balancing both the endorphinergic system with DPA and balancing the glutaminergic system with NAC together will optimize dopamine function and potentially induce required “dopamine homeostasis” in the long-term. Preceding this conceptualization, Blum et al. [8] had continuously added a small amount of L-Glutamine in the KB220z complex and had very good clinical outcomes. In similar terms, the single utilization of DPA has also failed.

## D-Phenylalanine (DPA) Failed Reports

DPA, alongside morphine, acetylsalicylic acid and zomepirac sodium, were assessed for their anti-nociceptive effects in monkeys (M. fascicularis) taught to auto-regulate nociceptive stimulation using a discrete-trial, aversive-threshold model [26]. Morphine sulfate created dose-dependent surges in harsh threshold, which were reversible following naloxone administration (12.5 or 25 micrograms/kg i.m.). D-Phenylalanine (500 mg/kg p.o.) made a minor growth in harsh threshold, which was not statistically significant and naloxone irreversible. Acetylsalicylic acid (200 mg/kg p.o.), unlike zomepirac sodium (200 mg/kg p.o.), in conjunction with D-phenylalanine (500 mg/kg), created a minor statistically significant increase in harsh thresholds [27]. These outcomes dispute the hypothesis that D-phenylalanine is liable for rising harsh thresholds via opiate receptor mechanisms encompassing increased enkephalin activity at synaptic loci. Earlier studies in rats and mice presented D-phenylalanine and acetylsalicylic acid as creating surges in nociceptive thresholds, which were naloxone reversible. The failure to discover opiate receptor mediated analgesia in a primate standard with confirmed opiate receptor selectivity and sensitivity may interfere with former research demonstrating an analgesic part for D-phenylalanine. Other clinical research has also failed to display a positive result on morphine pain resilient patients [26].

## Combining DPA and NAC in Combination: Detoxification of Opioid Addicts

In an unpublished pilot study, one of us (KB) combined both DPA and NAC along with other amino-acid precursors, Chromium salts and Rhodiola *rosea*. In seventeen opioid addicts undergoing detoxification, we show that the KB220z in a liquid nano was successfully able to reduce withdrawal symptoms and in fact, instead of detoxifying opioid addicts with the combination of buprenorphine/naloxone (Suboxone, Zubsolve), the utilization of KB220z liquid variant substituted for buprenorphine/naloxone not only in detoxification, but is also providing maintenance in 88.3 % of the studied patients [28].

## Dopamine Agonist Therapy: Changing the Recovery Landscape

In the right direction, compounds that are enkephalinase inhibitors affect dopamine release. For example, the enkephalinase inhibitor sodium nitroprusside (SNP) was found to induce dopamine increase in striatum dialysates of spontaneously mobile rats. This compound by itself has not yet been studied as an anti-craving substance [25]. Existing neurologic circuits, particularly the brain reward cascade and the dopamine D2 receptor, shed light on reward mechanisms affecting behavioral craving [29]. It was found that reduced expression of the D2R in the nucleus accumbens and hippocampus was correlated with greater seeking during signaled non-availability of the drug supporting the overall effect of neurotransmitter activity within the mesolimbic system, resulting in the “reward,” in which the NAc releases DA and then interacts with dopamine D1-D5 receptors [29]. Consequently, this “reward cascade” [1] involves serotonin release and subsequent stimulation of the hypothalamic release of enkephalin. This, in turn, acts to inhibit GABA in the substantia nigra, regulating the amount of dopamine released at the “reward site”: the NAc. It is established that under normal conditions in the NAc, DA maintains and controls our normal drives relating to pleasure and under chronic conditions, reverts to motivational “wanting” not “liking” [30]. In fact, dopamine has come to be known as the “pleasure molecule” or the “anti-stress molecule” [31]. Synaptic release of dopamine stimulates a number of receptors (D1-D5), leading to increased feelings of well-being and decline in stress. Positron emission tomography (PET) has demonstrated that substantially decreased levels of dopamine D2 receptors in alcohol and drug dependent subjects are relative to those levels in non-dependent individuals [32].

In animals, over expression of the dopamine D2 receptor via vector delivery of the D2 gene resulted in notable reduction of alcohol and cocaine consumption [33–35]. KB220Z, a dopamine agonistic agent, normalizes brain impairments, especially by potentially activating the release of brain dopamine at the reward site and thus, reducing excessive craving behaviors and induction of enhanced resting state functional connectivity [36].

It is established that after prolonged abstinence from drugs of choice, individuals will experience a more euphoric high, which can lead to relapse. This clinically observed “super sensitivity” might point toward the existence of genetic dopaminergic polymorphisms [37]. Paradoxically, it is interesting to note that bromocriptine, a dopaminergic agonist, causes an increase in brain reward activity in individuals who carry the DRD2 A1 allele in comparison to DRD2 A2 carriers [38]. Since A1 carriers, relative to A2 carriers, exhibit much lower D2 receptor density, A1 carriers should theoretically experience a reduced sensitivity to dopamine agonist activity [38]. Yet, it is observed that low D2 density corresponds to increased reward sensitivity with bromocriptine [39]. Furthermore, with chronic or long-term therapy of D2 agonists, there is a proliferation of D2 receptors in vitro [40]. However, in vivo studies show the opposite-a down regulation of D2 receptors after bromocyrtine administration [41]. The importance of utilizing amino-acid therapy may be explained by how dopamine is synthesized. L-amino acid decarboxylase undergoes striatal activity, which is associated with the A1 allele before forming dopamine.

Specifically, Laakso et al. [42] reported that the A1 allele corresponds to the increased activity of striatal L-amino acid decarboxylase in healthy Finnish subjects. They found that heterozygous carriers of the A1 allele (A1/A2; 10 subjects) had significantly higher [18%] [18F]-FDOPA uptake in the putamen than subjects without the A1 allele (A2/A2; 23 subjects). These results are evidence that the A1 allele corresponds to L-amino acid decarboxylase, which is a rate-limiting enzyme for trace amine synthesis and is present in the final step of dopamine synthesis. Lower D2 receptor expression, which precipitates decreased auto-receptor function, may explain this correlation and may suggest that dopamine and trace amine synthesis rate is higher in A1 allele carriers, a risk for all addictive behaviors [42]. It is our interpretation that carriers of the DRD2 A1 allele may have an interesting built-in protective mechanism waiting for amino-acid introduction such as L-phenylalanine and L-tyrosine (rate-limiting step in the synthesis of dopamine).

These genetic and non-genetic (epigenetic) effects may even last in future generations and could explain a better compliance of amino-acid therapy, especially in carriers of the D2 receptor deficient DRD2 A1 allele. We suggest that “dopamine agonist therapy,” such as KB220 variants, can reduce methylation and increase acetyl groups to enhance DRD2 expression, even in DRD2 A1 allele carriers [43]. This should then lead to increased DA function and reduction of drug and non-drug seeking behaviors and mortality [44].

Finally, Badgaiyan et al. [45] provided the most promising and clearest evidence: the status of brain dopamine varies with either resting state or during dopamine activation in human ADHD subjects. The take home message here is that since Attention Deficit Hyperactivity Disorder is a subset of RDS, these data reveal that there is lower dopamine at rest, which suggests a hypodopaminergic trait supporting our original hypothesis related to RDS and neurotransmission [36].

It is uncertain whether or not attention deficit hyperactivity disorder (ADHD) is a hypodopaminergic or a hyperdopaminergic or a disturbed glutamatergic [46] disorder. Diverse sets of data imply either the presence of a hyperactive or hypoactive dopamine system, since secondary approaches utilized in previous studies have reached inconsistent conclusions. Badgaiyan et al [45] directly measured the tonic and phasic dopamine release in ADHD subjects. The tonic release in both ADHD and healthy control subjects was measured and matched using a dynamic molecular imaging technique. The phasic release throughout the presentation of Eriksen’s flanker task was measured in the two individual groups using a single scan dynamic molecular imaging technique. In these trials, subjects were situated under a positron emission tomography (PET) camera and administered a dopamine receptor ligand 11C-raclopride intravenously. Following the injection, PET data were attained dynamically, while subjects either remained still (tonic release experiments) or completed the flanker task (phasic release experiments). PET data were evaluated to measure dynamic variations in ligand binding potential (BP) and other receptor kinetic parameters. The analysis exposed that at rest, the ligand BP was considerably greater in the right caudate of ADHD subjects, implying decreased tonic release. During task performance, drastically lowered ligand BP was seen in the same region, representing increased phasic release. In ADHD, dopamine tonic release is reduced, and the phasic release is augmented in the right caudate. By typifying the nature of dysregulated dopamine neurotransmission in ADHD, the outcomes clarify prior findings of decreased or augmented dopaminergic activity.

## Conclusion

It is our contention that the novel combination of DPA and NAC by itself and or in combination with KB220Z will provide a safe and effective natural way to induce dopamine homeostasis for the millions of sufferers of RDS. The simple message is that independent of either genetic antecedents or epigenetic impacts, this is a combination whereby DPA can effect dopamine release at the NAc via an endorphinergic mechanism, affecting GABA transmission and whereby NAC can also affect the glutaminergic system by inducing homeostasis together, creating a balancing effect that should promote required “dopamine homeostasis” in a synergistic manner. The goal is to convert the unhappy brain to a happy one by controlling the balance of GABA, serotonin and enkephalins.
